# Photoacoustic Spectroscopy-Based Detection for Identifying the Occurrence and Location of Laser-Induced Damage Using a Laser Doppler Vibrometer

**DOI:** 10.3390/s25216643

**Published:** 2025-10-30

**Authors:** Katsuhiro Mikami, Ryoichi Akiyoshi, Yasuhiro Miyasaka

**Affiliations:** 1 Faculty of Biology Oriented Science and Technology, Kindai University, Kinokawa 649-6493, Wakayama, Japan; 2 Graduate School of Biology Oriented Science and Technology, Kindai University, Kinokawa 649-6493, Wakayama, Japan; 3 Kansai Institute for Photon Science, National Institutes of Quantum Science and Technology, Kizugawa 619-0215, Kyoto, Japan

**Keywords:** laser-induced damage, photoacoustic spectroscopy, detection, vibration, laser Doppler vibrometer

## Abstract

We present a photoacoustic spectroscopy (PAS)-based method using a laser Doppler vibrometer (LDV) for real-time detection of laser-induced damage (LID) in optical components. By measuring audible frequency surface vibrations, the method enables remote, non-contact, and sensitive detection. Experiments with various dielectric optics (slide glass and single-layer coatings) and pulse durations (7 ns and 360 ps) of an Nd:YAG laser (wavelength of 1064 nm) showed detection accuracy comparable to microscopy. Vibration spectra correlated with natural modes calculated by finite element modeling, and vibrations according to the detecting location were observed. The method remained effective under typical mounting conditions, demonstrating its practical applicability. This PAS-LDV approach offers a promising tool for in situ monitoring of LID in high-power laser systems.

## 1. Introduction

Early detection of laser-induced damage (LID) is essential for maintaining the stability of high-power laser systems. In this study, we propose a photoacoustic spectroscopy (PAS)-based detection approach using a laser Doppler vibrometer (LDV) to realize non-contact, real-time monitoring of LID. LID is defined as an irreversible destruction phenomenon occurring in optical components. Once such damage takes place, stable operation of laser systems can no longer be maintained. Consequently, the laser output must be limited to a level below the laser-induced damage threshold (LIDT). The LIDT is generally defined in terms of energy density, as localized concentration of energy in space tends to trigger laser damage. Therefore, fluctuations or disturbances in the performance of laser components can cause changes in the beam profile, occasionally leading to unexpected damage events. Once initiated, laser damage often expands at the affected site, resulting in what is known as functional damage. Ultimately, the enlarged damage area degrades the laser beam profile, potentially inducing secondary damage in downstream optical elements [1]. For this reason, repair or replacement strategies are typically formulated based on the size and number of damage sites. Thus, a highly sensitive, real-time, in situ detection method for laser-induced damage is essential for the reliable operation and maintenance of laser systems [2].

Optical components such as mirrors, windows, and lenses incorporated in laser systems are typically coated with dielectric or metallic films to achieve the desired optical properties. Laser-induced damage tends to occur preferentially in regions with lower LIDT values. As a result, damage is most likely to initiate at the outermost surface, such as dielectric thin films or metallic coatings, where the LIDT is generally lower than that of the bulk dielectric substrate or internal materials [3]. Detection of such surface laser damage is typically performed through microscopic surface inspection. In recent years, efforts have been made to enhance this approach by integrating various illumination techniques with deep learning methods [4,5,6,7]. However, these advanced image-based inspections involve processing large volumes of image data containing two-dimensional spatial and intensity (brightness) information, thereby requiring substantial computational resources.

To address the limitations of microscopy, various alternative detection methods for laser-induced damage have been actively investigated. In dielectric optical components, the dominant laser damage mechanism at nano-second pulse widths is known to be avalanche ionization. By fabricating electrodes on the optical surface, researchers have attempted to detect the precursor to damage—namely, the generation of faint electrical currents resulting from initial stages of avalanche ionization [8]. However, this requires the formation of metal electrodes on the optical element surface, which poses challenges for in-line use.

Among non-microscopic methods, PAS has emerged as a promising technique for laser damage detection. The principle of PAS lies in the conversion of absorbed laser energy within an optical component into thermal energy, which in turn generates elastic (acoustic) waves. Several studies have explored the detection of these acoustic waves using ultrasonic transducers, piezoelectric sensors, microphones, or position sensors [9]. The photothermal deflection technique using a position sensor, for instance, attempts to detect early signs of damage by monitoring changes in the pointing direction of a probe beam, which are caused by refractive index variations induced by localized thermal energy [10,11,12,13]. However, the detection accuracy of PAS-based methods remains inferior to that of optical microscopy [14]. In the case of ultrasonic transducers and piezoelectric sensors, measurements are typically taken from the rear surface or locations distant from the actual damage site. As a result, the effect of wave attenuation becomes non-negligible and compromises sensitivity. When using microphones, additional equipment such as a photoacoustic cell is generally required, and the detection is limited by the inherent sensitivity to minute air vibrations. These factors impose fundamental limitations on the measurement principle itself.

Recently, increasing attention has been directed toward a novel approach in PAS that focuses on measuring vibrations in the audible frequency range, rather than traditional shock waves in the ultrasonic domain. This technique employs an LDV to detect surface vibrations of the sample [15]. This can be described as hammering testing replaced with laser technology. For example, this technology has made it possible to perform hammering testing for internal defects in concrete [16,17,18]. This technology uses active excitation with a laser, but it is also possible to observe passive phenomena. By utilizing laser-based vibration measurement, the method enables remote, non-contact sensing directly on the surface of the optical component when causing LID. Moreover, this approach targets the detection of sample-specific vibrational modes that persist for several milliseconds and primarily occur in the audible frequency range. As a result, it eliminates the need for phase-sensitive detection or high temporal resolution measurements, which are typically required in ultrasonic-based PAS methods.

In this study, we experimentally demonstrated LID detection based on the sample’s intrinsic vibrations measured by LDV in the audible frequency range. Damage tests were conducted on dielectric optical components mounted in various types of holders, using two types of near-infrared laser pulses with different pulse widths. The accuracy of damage detection using laser-induced vibrations was compared with that of conventional microscopic inspection. Furthermore, by analyzing the vibration data, we verified the improvement in detection accuracy and the possibility of identifying the damaged area, which are reported in this paper.

## 2. Materials and Methods

**Figure 1:**
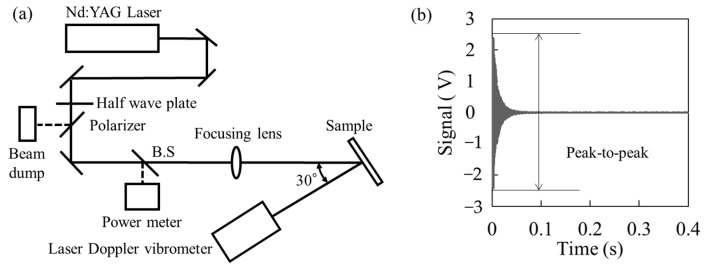
Experimental details of (**a**) setup and (**b**) typical obtained signal.

Figure 1 shows the experimental details of the proposed PAS scheme. Figure 1a shows an experimental setup. Two kinds of pulse width (7 ns and 360 ps [19]) with a wavelength of 1064 nm were used from different Nd:YAG laser systems. The pulse energy was adjusted by a half-wave plate and polarizer, then the value was measured by an energy meter. The laser pulse was focused by lens (f = 300 mm at 7 ns pulse width, f = 407 mm at 360 ps pulse width), and the typical diameters of the spot sizes at normal incidence were 370 μm at 7 ns and 170 μm at 360 ps, respectively. The experimental sample was irradiated laser pulse with 30 deg incident angle, and the occurrence of LID was monitored. The LDV was positioned directly opposite the experimental sample and irradiated the same site as the Nd:YAG laser to detect the vibrations associated with LID generation. The LDV was set with a 20 kHz low-pass filter to obtain velocity information on the sample surface in the audible frequency range, and the output voltage signal (sensitivity 5 mm/s/V) was recorded by an oscilloscope. This low-pass filter is effective for limiting the frequency range to the audible spectrum when analyzing with broadband vibration excitation spanning the ultrasonic range, generated by nano-second pulse width impulse excitation. The high-pass filter is particularly effective for removing environmental noise components occurring below 100 Hz. In this study, it was unnecessary because sufficiently large vibrations were obtained during LID generation. It is anticipated to be necessary for future precise measurements, such as those involving the measurement of minute absorptions in optical elements. The LID was detected by LDV and plasma emission at the same time with laser irradiation, and microscope observation was performed after the damage test. The LIDT evaluated by microscopy and plasma emission was defined as the maximum fluence without LID occurrence. Figure 1b shows the typical voltage signal output from the LDV when LID occurred. The occurrence of LID and its LIDT were defined by the appearance of a peak-to-peak value above its base signal and the maximum fluence without LID, respectively. Thus, by interpreting the acoustic waves generated through laser irradiation as surface vibrations, PAS-based detection of laser-induced damage can be realized using a laser Doppler vibrometer.

Three kinds of experimental samples—Ta_2_O_5_ single-layer coating, dielectric multilayer coating mirror, and slide glass—were used. The Ta_2_O_5_ single-layer coating was prepared on a silica glass substrate with a design thickness of a quarter wavelength of 1064 nm, and the dielectric multilayer coating mirror was prepared by a typical commercial product. The slide glass sample, which had a simple rectangular form, was used to evaluate the influence of natural vibration modes. The sizes of the Ta_2_O_5_ single-layer coating, dielectric multilayer coating mirror, and slide glass were 30 mm in diameter and 1 mm thick, 25.4 mm in diameter and 6 mm thick, and 76 × 26 mm in diameter with 1 mm thickness, respectively. The LIDTs defined with microscopy observation of the Ta_2_O_5_ single-layer coating, dielectric multilayer coating mirror, and slide glass were measured by 1-on-1 testing with a 7 ns pulse width as 8.1 J/cm^2^, 2.4 J/cm^2^, and 11 J/cm^2^, respectively. The 1-on-1 test was performed by irradiating a single laser pulse at each point with 1 mm spacing while varying the fluence, regardless of whether damage occurred. For each sample, approximately 70 irradiation points were tested, and the LIDT was defined as the fluence one step lower than the minimum fluence at which visible damage was observed. In this study, the evaluation of LID detection accuracy was carried out based on the analysis of individual irradiation data obtained from the 1-on-1 test.

## 3. Results

**Figure 2:**
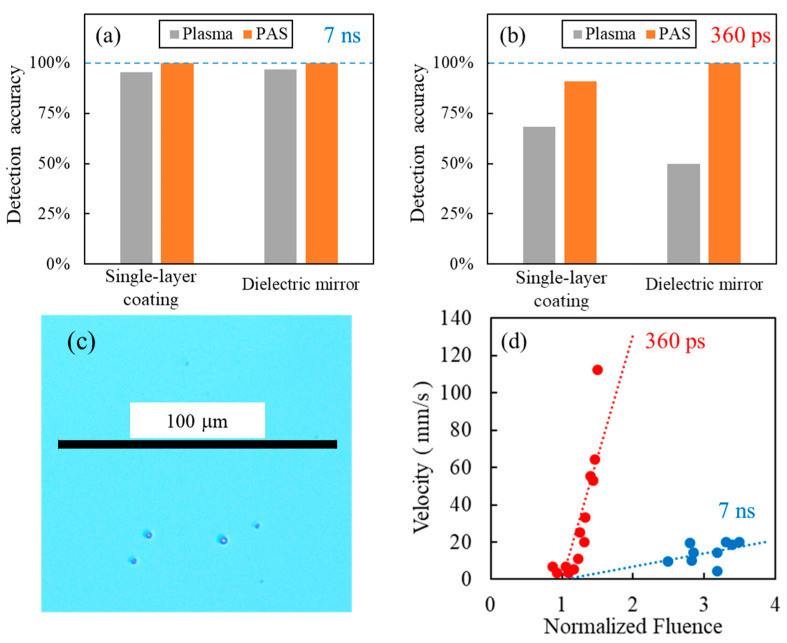
PAS-based LID detection performance. The accuracies at (**a**) 7 ns and (**b**) 360 ps measurement compared with microscopy observation. The (**c**) microscope image of the smallest size in the observed LID and (**d**) sensitivity of the PAS-based detection scheme in single-layer coating at different pulse widths.

Figure 2 shows the performance of the proposed PAS-based LID detection scheme. The detection accuracy of PAS-based scheme was better than that obtained by plasma emission and comparable with microscope observation, regardless of pulse width as shown in Figure 2a,b. A microscope image of the smallest LID detected under 7 ns laser pulse irradiation is shown in Figure 2c. The damage caused by the minimum fluence in this experiment showed a damage shape consisting of scattered LIDs on the surface, with a diameter of about 5 μm. LIDs much smaller than the laser irradiation spot size of 300 μm in diameter could be detected. At this time, the peak-to-peak value of the voltage signal was about three times higher than the baseline, which was sufficiently detectable. The vibration velocity used to determine the LIDT for the single-layer coating sample in this experiment was 86 μm/s, which is equivalent to a displacement of several tens of nanometers. The velocity values were derived from the output voltage using the calibration coefficient of the laser Doppler vibrometer (sensitivity: 5 mm/s/V), as described in the Methods section. The corresponding displacement was approximately estimated by integrating the velocity waveform over time. The output signal from the LDV indicated velocity on the experimental samples, and the maximum variation, i.e., peak-to-peak value, represented the sensitivity to detect LID presence. Figure 2d shows the relationship between irradiation fluence normalized with LIDTs and the maximum variation in velocity in the case of the Ta_2_O_5_ single-layer coating. The shorter pulse width provided a specific increase in velocity with increasing irradiation fluence, regardless of the experimental samples. The measurement principle of PAS is that light energy is absorbed and converted into thermal energy within the material, which is then finally converted into kinetic energy due to thermal expansion. Therefore, a shorter pulse width is less affected by thermal diffusion, and PAS measurements can be achieved efficiently, resulting in high sensitivity.

## 4. Discussion

**Figure 3:**
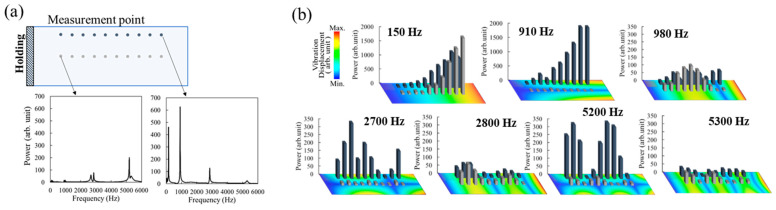
Relationship between measurement points and the vibrational spectra. (**a**) shows typical difference in spectra at different points and (**b**) shows the spectral powers at each measurement points for different natural vibrational modes.

The LID detection scheme using PAS detects the instantaneous velocity variation at the time of laser irradiation. Similar decaying vibration behaviors following laser excitation have been reported in previous photoacoustic and laser-induced vibration studies on solid materials [9]. On the other hand, the vibration continues for hundreds of milliseconds while attenuating, as shown in Figure 1b. This is due to the natural vibration determined by the shape and mechanical properties of the material. Figure 3a shows an example of the vibration spectrum obtained by fast Fourier transform (FFT) of the time-domain velocity waveform obtained by LDV when LID was generated at different laser irradiation sites on the slide glass. To clarify the effect of the natural vibration, the slide glass was fixed like a cantilever and the fluence was adjusted to twice the LIDT. It was revealed that the vibration spectra were completely different depending on the LID site. To consider this vibration spectrum, the natural vibrations of the experimental sample were calculated by the finite element method (FEM). The finite element analysis was performed using Autodesk Fusion 360 to conduct a steady-state modal vibration analysis. The mesh size was determined as 3% of the total sample dimension to ensure adequate accuracy while maintaining computational efficiency. As shown in Figure 3a, the sample was modeled as a cantilever beam, with the 2 mm region at one end completely fixed as the boundary condition during the simulation. The relationship between the peak values of the vibration spectra and the natural vibration is shown in Figure 3b. The color map on the bottom indicates the calculation results, and the warmer the color, the larger the displacement of the natural vibration. It was found that there was a correlation between the displacement of the natural vibration and the peak power of FFT analysis in all vibration modes, from the fundamental vibration mode (150 Hz) to the higher vibration mode (5200 Hz). This correlation between the measured vibration modes and FEM-predicted natural frequencies is consistent with prior works applying modal analysis to mechanical structures [17].

In this experiment, the Nd:YAG laser used for inducing the LID and the LDV were irradiated at the same location for comparison with the FEM calculation results, and the demonstration showed good agreement. Additionally, a measurement scheme with fixed irradiation position for the LDV is desirable for easy use in actual operations. Since the LID generation position in the laser irradiation area is unknown, it is generally considered most effective to measure the antinode of the fundamental vibration mode. Table 1 shows the vibration intensity increase ratio of the natural vibration mode (150 Hz), from the evaluation position closest to the free end to the evaluation position closest to the fixed end, in the test shown in Figure 3a. The two rows of measurement positions as shown in Figure 3a were defined as Upper and Center, and verification was performed using the calculated values by FEM and the experimental values of the spectrum peak obtained by FFT. At the Center, the calculated values and experimental values for the increase in vibration intensity were consistent. On the other hand, the experimental value obtained a vibration increase rate that exceeded the calculated value at the Upper. This is thought to be because there are many high-order vibration modes with the largest displacement on the free end side of the Upper, which results in a large displacement and a high vibration peak in the FFT analysis. These results suggest that the LDV can efficiently detect LID by conducting fixed-point observations at locations where the largest displacement is predicted, including not only the fundamental vibration mode but also higher-order vibration modes.

Identifying the LID position relative to the laser irradiation surface of the optics is not necessary to determine whether to shut down a laser system during simple use. However, in actual system operations, it is essential information required for confirming the actual LID condition during system maintenance and/or optical element repair work. In the LID detection scheme using PAS, it is possible to predict the location of LID from two schemes. The first is by understanding the relationship between the natural vibration modes of the material and the impulse position in the vibration spectrum. The priority excitation vibration is selected according to the impulse position. This analysis scheme requires further investigation in the future. The second scheme combines information about the vibrational phase. This is like the acoustic emission method used in the field of non-destructive testing and shows that adding vibration phase information can provide a similarly high-precision analysis [20,21,22].

**Figure 4:**
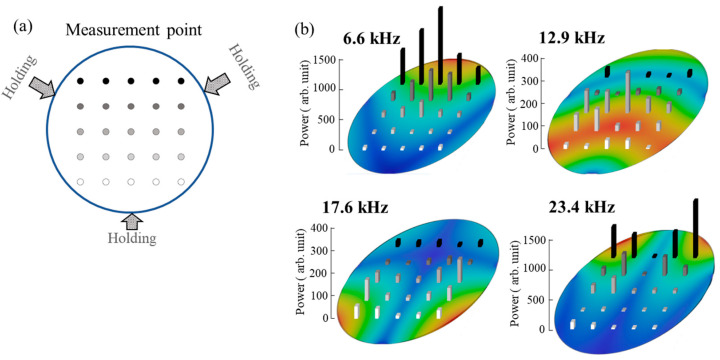
Evaluation of relationship between vibrational power and natural vibrational modes with a typical mirror holder: (**a**) holding scheme and (**b**) results.

The evaluation results of the cantilever-like fixation shown in Figure 3 are different from the typical holding method of optical elements. The proposed method was verified using a three-point support mirror holder as a general holder of optics. Figure 4a shows the fixing condition and laser irradiation position in the evaluation using a single-layer coating. As a result of this consideration, a correlation similar to that of the result in the slide glass experiment was obtained, as shown in Figure 4b. The finite element modeling for Figure 4 was performed in the same way as that for Figure 3. The optical component surface was constrained by fully fixing the vertex positions of an equilateral triangle with a width of 2 mm, which served as the boundary condition in the vibration analysis. Since the fixing positions in an actual mirror holder are diverse, we compared the results with those of an FEM analysis using a simple three-point support at equally spaced positions. In other words, this PAS-based scheme is also effective for general mirror holders, and it is expected that lens holder with circumferential fixation will be easier to detect because it shows a simpler natural vibration mode.

In this study, LID detection was performed using the peak-to-peak value of the time-domain voltage waveform, and the occurrence location of LID was estimated from the frequency spectrum obtained through FFT analysis. Since these data are one-dimensional, the computational load for data processing is minimal, enabling fast and straightforward implementation. This approach is particularly effective for continuously operated, high-repetition laser systems. However, FFT analysis inherently loses time-domain information, making it unsuitable for evaluating vibration phase or transient responses. By applying time–frequency analysis methods such as wavelet transform or short-time Fourier transform (STFT), it is possible to construct spectrograms that utilize both vibration and temporal information. Although this increases data volume and analytical resource requirements, it can provide more detailed information for precise localization of LID events [23,24,25,26]. This method is considered particularly effective for large-scale, low-repetition-rate, high-power laser facilities.

## 5. Conclusions

In this study, we demonstrated an LID detection scheme based on PAS using LDV. By focusing on audible frequency surface vibrations generated during LID events, the proposed method enables remote, non-contact, and highly sensitive detection of damage in dielectric optical components. The detection accuracy was found to be comparable to microscopic observation and superior to conventional plasma emission methods. Furthermore, analysis of the vibration spectrum revealed that the measured vibration modes matched the natural frequencies of the specimen calculated by the finite element model, which provides insight into the location of the damage. This capability was validated across different sample types and mounting conditions, including typical optical holders. Compared to conventional PAS that use ultrasound, these results demonstrate that utilizing vibrations in the audible range makes it possible to realize an inexpensive and practical real-time monitoring system for laser systems. This result is expected to contribute to the reliability and maintenance strategies of facilities, especially for large, high-power laser systems that are at risk of LID and require long maintenance periods.

## Figures and Tables

**Table 1:** Increase in vibration strength of mode 1 (150 Hz) as shown in Figure 3b at the free end compared to the fixed end.

Line	Calculation or Experiment	Increase Rate
Upper	FEM	15.1
	Spectrum	34.8
Center	FEM	14.3
	Spectrum	13.7

## Data Availability

The datasets are available from the corresponding author on reasonable request.
